# Action Postponing and Restraint Varies among Sensory Modalities

**DOI:** 10.3390/brainsci12111530

**Published:** 2022-11-11

**Authors:** Koyuki Ikarashi, Daisuke Sato, Genta Ochi, Tomomi Fujimoto, Koya Yamashiro

**Affiliations:** 1 Major of Health and Welfare, Graduate School of Niigata University of Health and Welfare, Niigata City 950-3198, Niigata, Japan; 2 Sports Physiology Laboratory, Department of Health and Sports, Niigata University of Health and Welfare, Niigata City 950-3198, Niigata, Japan; 3 Institute for Human Movement and Medical Sciences, Niigata University of Health and Welfare, Niigata City 950-3198, Niigata, Japan; 4 Research Fellowship for Young Scientists, Japan Society for the Promotion of Science, Tokyo 102-0083, Japan

**Keywords:** action postponing, action restraint, event-related potential (ERP), Go/no-go task, sensory modality

## Abstract

Proactive inhibition is divided into two components: action postponing (AP), which refers to slowing the onset of response, and action restraint (AR), which refers to preventing the response. To date, several studies have reported alterations in proactive inhibition and its associated neural processing among sensory modalities; however, this remains inconclusive owing to several methodological issues. This study aimed to clarify the differences in AP and AR and their neural processing among visual, auditory, and somatosensory modalities using an appropriate experimental paradigm that can assess AP and AR separately. The postponing time calculated by subtracting simple reaction time from Go signal reaction time was shorter in the visual modality than in the other modalities. This was explained by faster neural processing for conflict monitoring induced by anticipating the presence of the No-go signal, supported by the shorter latency of AP-related N2. Furthermore, the percentage of false alarms, which is the reaction to No-go signals, was lower in the visual modality than in the auditory modality. This was attributed to higher neural resources for conflict monitoring induced by the presence of No-go signals, supported by the larger amplitudes of AR-related N2. Our findings revealed the differences in AP and AR and their neural processing among sensory modalities.

## 1. Introduction

Response inhibition (RI) is the ability to inhibit automatic actions and inappropriate responses and supports behavioral flexibility in changing environments [1]. This function consists of proactive and reactive inhibition [2], which are important components of executive control and play a crucial role in everyday life. Proactive inhibition includes action postponing (AP) and restraint (AR) [3]. AP refers to the postponement of movement initiation when anticipating having to hold or stop [4,5] and AR refers to the withholding of action for applying an external signal when forecasting in advance of an overt need to hold [6,7]. Reactive inhibition, also termed action cancellation (AC), is the ability to cancel planned or already-initiated action based on unexpected cues [8,9]. RI can be commonly studied using the Go/No-go task (GNT) and Stop-signal task (SST).

In both proactive and reactive inhibition, the ability to detect changes in sensory information plays a key role in RI. Proactive inhibition is triggered by both endogenous and exogenous sensory signals with maintaining the information about how and when inhibition should occur and reactive inhibition is modulated by unexpected exogenous sensory signals [10,11,12]. Additionally, previous neuroimaging evidence indicates that not only a “modality-common” neural substrate, but also a “modality-specific” neural substrate are related to RI [6]. Therefore, the sensory modality of signals triggering an inhibition process may affect RI. Our previous study of differences in reactive inhibition among sensory modalities revealed that AC does not differ among sensory modalities, while AC-induced AP varies [13], suggesting that sensory modality may exert different effects according to the type of RI. Furthermore, although several studies have investigated whether sensory modality affects proactive inhibition, especially AR, this issue continues to be investigated [6,14,15,16]. Moreover, no study has examined the effect of sensory modality on AR-induced AP. This is due to the following methodological issues, although RI can be commonly studied using the GNT. The first is the inconsistent method for assessing AR. Some studies used reaction time for the Go signal (Go-RT) [16,17], while others used several types of false alarms (FAs) [14,15]. Assessing AR by the latter method is considered superior since AR refers to whether withholding the action or inhibition should be implemented [12]. Second, previous studies have not evaluated AP and AR separately. Basically, AP should be evaluated by how long the RT is slowing by anticipating the possibility of applying the No-go signal [5]. Therefore, it is better to assess AP based on the time difference between the Go-RT with and without the No-go signal. Third, most studies have not used the GNT paradigm considering stimulus-response compatibility (SRC), which refers to the phenomenon in which some mappings between a particular set of stimuli and responses allow for better performance than other mappings [18,19]. Several studies have found that the SRC effect in the visual and auditory GNT is similar to that in choice reaction tasks [16,20]. Therefore, the GNT paradigm should be used with a consistent set of stimuli and responses when investigating the differences in AP and AR among the sensory modalities. Finally, no research has examined the differences in AP and AR among the three modalities in the same participants. So far, the differences between just two modalities have been examined [6,14,15,16]. Based on these results, a novel experimental paradigm to reveal the differences in AP and AR among the three modalities in the same participants is required to elucidate the proactive inhibition-related unique and common neural substrates across sensory modalities.

The event-related potential (ERP) can be used to investigate proactive inhibition-related neural processing. Previous ERP studies have mainly reported two major components associated with proactive inhibition in the GNT. The first is No-go-N2, a negative deflection at the frontal-midline sites that peaks around 200–400 ms following stimulus onset, which reflects premotor inhibitory processes [15,21] and conflict monitoring [22,23]. The second component is No-go-P3, the subsequent centroparietal positive shift; it is at its maximum at approximately 300–600 ms following stimulus onset, which reflects mirror inhibitory processes or the evaluation of successful inhibition [24,25,26]. Both of these components are usually evaluated by subtracting the Go signal-triggered ERP (Go-ERP) from the No-go signal-triggered ERP (No-go-ERP) (No-go minus Go), and have been reported as AR-related neural processing when assessed by FA [15,27,28]. However, an appropriate assessment method for AP-related neural processing has not yet been developed. AP refers to a delayed response to a Go signal and is a distinct inhibitory function of AR, which refers to the withholding of action [2]. Since AP occurs by anticipating the possibility of applying the No-go signal in the GNT [5], AP-related neural processing can be evaluated by the subtracted waveform between the Go signal-triggered ERP with and without the No-go signal (Go-ERP in the GNT minus simple reaction task [SRT]-ERP). Owing to these methodological issues, the differences in AP-related neural processing among the three modalities remain unclear. Therefore, to elucidate the proactive inhibition-related unique and common neural substrates across sensory modalities, it is necessary to examine the differences among the three modalities by separately assessing AP- and AR-related neural processing.

The present study aimed to clarify the differences between AP and AR and their neural processing among the visual, auditory, and somatosensory modalities. Proactive inhibition, which includes AP and AR, is goal-directed and should be mediated by working memory (WM) to handle information about how and when inhibition should be driven [12]. Several studies have reported higher visual WM compared to other sensory modalities [29,30], which could enable faster and higher neural processing in the visual modality. Therefore, we hypothesized that the visual modality would drive more effective AR (lower false alarms [FAs]) with shorter AP (shorter postponing time [PT]) than the other modalities. The factors underlying shorter PT are presumed to be a shorter latency of AP-related N2 in the visual modality, which is calculated by subtracting the SRT-ERP from the Go-ERP in the GNT, since N2 latency is an index of neural efficiency for driving proactive inhibition in the GNT [17]. Additionally, the lower FA might be explained by the larger AR-related N2 in the visual modality, which is calculated by subtracting the Go-ERP from the No-go-ERP in the GNT; previous research has demonstrated that a larger N2 amplitude results in precise responses owing to higher neural activity for conflict monitoring [31,32].

## 2. Materials and Methods

### 2.1. Participants

Twenty-one healthy right-handed adults (11 men and 10 women) with normal or corrected-to-normal vision and audition participated in this study. The sample size was calculated and determined using Superpower [33], which indicated that a sample of 20 would be sufficient for 85% power and an effect size of 0.25. The participants with a history of neurological or psychiatric disorders and female participants using hormonal contraceptives (oral contraceptives) were excluded. Informed consent was obtained from all participants. All the experiments conformed to the Declaration of Helsinki and the present study was approved by the ethics committee of Niigata University of Health and Welfare, Japan (18828-220513). All the female participants underwent the experimental procedure during their follicular phase (days 1–4 after menstruation) when the effect of sex steroid hormones are less, given that the previous study reported that the RI and associated neural activity showed fluctuations during the menstrual cycle and were affected by menstruation-related symptoms [34]. All the experiments were conducted between 9 AM and 1 PM to account for the circadian fluctuations.

### 2.2. Procedure

**Figure 1:**
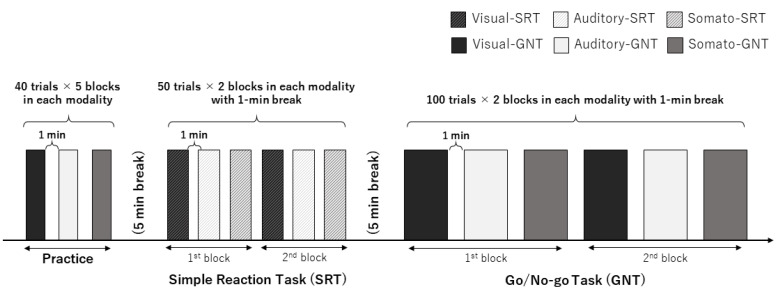
Experimental procedure in the present study. First, the participants were provided practice blocks (40 trials × 5 blocks) of the GNT in the visual, auditory, and somatosensory modalities with a 1 min break. After the practice block, SRT was conducted in two blocks for each modality with a 1 min break. Finally, the participants performed two blocks of the GNT consisting of 100 trials per 1 block for each modality. SRT, simple reaction task; GNT, Go/No-go task.

The experimental procedure is illustrated in Figure 1. The participants underwent an SRT and GNT using visual, auditory, and somatosensory stimuli termed visual, auditory- and somato-SRT and GNT, respectively. One block in the SRT consisted of only 50 Go trials. One block in the GNT was 100 trials (50 Go trials and 50 No-go trials). Prior to starting the main experiment, all the participants were provided with five practice blocks (one block = 40 trials, total trials = 200) of each GNT according to a previous study [17] to prevent the learning effect on the results. The SRT session consisted of two SRT blocks for each modality. SRTs using each modality were performed at 1 min breaks. The GNT session was divided into two GNT sessions with a 5 min break to avoid fatigue, and one GNT session consisted of one GNT block for each modality with a 1 min break. The order of the modalities in practice, SRT, and GNT sessions was counterbalanced across the participants. Both the SRT and GNT were performed using a custom-order program (Medical Try System Co., Ltd., Tokyo, Japan), which was controlled by a PC, as described in our previous study [13]. The visual stimulus (white arrows) was applied using a custom-order light-emitting diode (LED) panel (MTS207642-01785, Medical Try System Co., Ltd., Tokyo, Japan) to prevent signal delay. Auditory and somatosensory stimuli were applied using earphones (YE- 103 J, Medical Try System Co., Ltd., Tokyo, Japan) and ring electrodes (FINGER ELECTRODE NM-451B, NIHON KODEN Co., Tokyo, Japan), respectively.

### 2.3. Simple Reaction Task (SRT)

**Figure 2:**
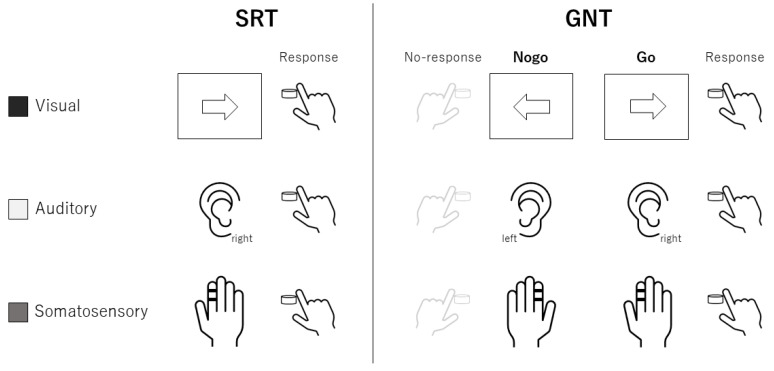
SRT and GNT paradigms in each modality. See text for details. SRT, simple reaction task; GNT, Go/No-go task.

Figure 2 illustrates the SRT paradigm. Participants were instructed to place their right and left index fingers on the right and left buttons, respectively. In the visual-SRT, the participants were instructed to press the right button as fast as possible when the right white LED arrow was presented for 1000 ms. In the auditory- and somato-SRT, the participants were instructed to press the right button when the right 1000 Hz (80 dB) pure tone for 500 ms and electrical stimuli (ES) with a pulse width of 200 µs were applied. The somatosensory stimuli were applied at 2.5 times the participant’s sensory threshold, which elicited no unpleasant sensations or pain. In each block, all the stimuli were randomly presented between 2.5 s and 3.5 s inter-trial intervals.

### 2.4. Go/No-Go Task (GNT)

The GNT paradigm is illustrated in Figure 2. Similar to the SRT, the participants were instructed to place their right and left index fingers on the button following each trial. The participants were instructed to press a button corresponding to the Go signals as quickly and precisely as possible with their right index finger. In contrast, the participants had to withhold their responses when a No-go signal was presented in the No-go trials. Considering the Go trials for each modality, the Go signals were presented in the same manner as the SRT protocols: right white arrow LED on the right side of the panel, 1000 Hz pure tone to the right ear, and ES to the right index finger. For the No-go trials, the No-go signals were left white arrow LED on the left side of the panel, 1000 Hz pure tone to the left ear, and ES to the left index finger in visual, auditory, and somatosensory modalities, respectively. The Go and No-go signals were randomly delivered with an ISI of 2.5 s to 3.5 s.

### 2.5. Behavioral Analysis

AP was assessed using the PT, which is the difference between the mean reaction time (RT) in the SRT (simple reaction time: S-RT) and Go trials (Go reaction time: Go-RT). The AR was assessed using the percentages of the FA (%FA) which is the reaction to the No-go signals.

### 2.6. Electroencephalogram (EEG) Recording and Analysis

The setup for EEG recording and offline analysis was employed according to our previous study, which measured the EEG activity during RI [13]. Continuous EEG was recorded from nine affixed electrodes (Fz, F3, F4, Cz, C3, C4, Pz, P3, P4), with those on the mastoids (M1–M2) as the recording reference, based on the 10–20 system during both SRT and GNT. EEG and electrooculograms (EOG) were recorded at a sampling rate of 2.5 kHz and filtered with a bandpass of 0.1–100 Hz and a notch of 50 Hz using a Brain Products amplifier system (Brain Products GmbH, Germany) and BrainVision Professional Recorder (Brain Products GmbH). All the electrode impedances were maintained below 5 K Ω. The EOG placed on the bilateral external canthi and the left infraorbital and supraorbital areas were simultaneously recorded with the EEG recordings to eliminate the artifacts attributed to eye blinks. EEG data were analyzed using BrainVision Professional Analyzer 2 (Brain Products GmbH), and EEG eyeblinks and moving artifacts were eliminated using an independent component analysis. For the offline analysis, a 0.1–30 Hz bandpass filter was applied to the continuous EEG data with a downsampling of 500 Hz. The EEG data that were epoched from 100 ms prestimulus to 500 ms post-stimulus corresponding to the Go signal in the SRT and GNT (Go trials), and the No-go signal in the GNT (No-go trials) were extracted and corrected using the prestimulus baseline. The epochs contaminated with artifacts exceeding ±100 μV were excluded from further analysis.

**Figure 3:**
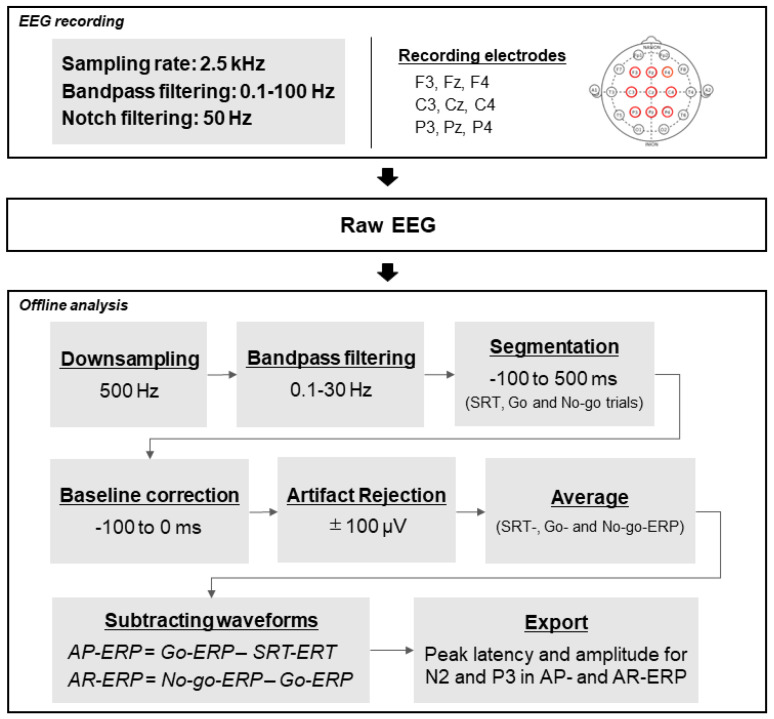
The flowchart for pre-process and analysis. AP, action postponing; AR, action restraint; ERP, event-related potential.

We extracted the Go signal-locked waveforms in the SRT and the Go and No-go signal-locked waveforms in the GNT, which were termed SRT-, Go-, and No-go-ERP, respectively. We calculated the waveform difference by subtracting the SRT-ERP from the Go-ERP (AP-ERP) to determine the neural processing related to AP. Additionally, we subtracted the Go-ERP from the No-go-ERP in the GNT (AR-ERP) to examine the neural processing associated with the differences in the AR. For these ERPs, N2 latencies and amplitudes were measured at Fz, F3, and F4 as the maximum negative values in the time window of 200–400 ms [14,35]. P3 latencies and amplitudes were measured at Cz and Pz as the maximum positive values in the time window of 300–500 ms [7,14]. The flowchart for pre-process and analysis is presented in Figure 3.

### 2.7. Data Analysis and Statistics

The behavioral data obtained from the SRT (S-RT), GNT (Go-RT, %FA), and PT in each modality were averaged. Parametric data (distribution confirmed by the Shapiro–Wilk test) were entered into a one-way repeated-measures analysis of variance (ANOVA) with “modality” (visual, auditory, and somatosensory) as the within-subject factor. Nonparametric data were analyzed using the Friedman test to compare the modalities; “modality” (visual, auditory, and somatosensory) as the within-subject factor.

The neurophysiological data, which are the latency and amplitude of the N2 and P3 components in the AP-ERP (AP-N2 and AP-P3) and AR-ERP (AR-N2 and AR-P3), were averaged for each modality. Parametric data were entered into two-way repeated-measures ANOVA with “modality” and “electrode” (N2; Fz, F3 and F4, P3; Cz and Pz) as the within-subject factors. Nonparametric data were analyzed using the Friedman test to compare the modalities, and the SRT, Go, and No-go-ERP components in each modality.

In all the analyses using repeated-measures ANOVA, the Greenhouse–Geisser correction was used to correct for non-sphericity if necessary; Bonferroni’s post hoc tests were used for the pairwise comparisons. For the nonparametric data, Wilcoxon’s signed-rank test and Bonferroni’s inequality were used for the pairwise comparisons. Spearman correlation analysis was performed to assess the relationship between the behavioral and neurophysiological data, and Bonferroni’s inequality was used to prevent type 1 errors. Statistical significance was set at *p* < 0.05. Data were analyzed using SPSS version 27 (IBM Corp., Armonk, NY, USA). All the data are expressed as the mean ± standard error of the mean (SEM).

## 3. Results

### 3.1. Performance of SRT and GNT

**Figure 4:**
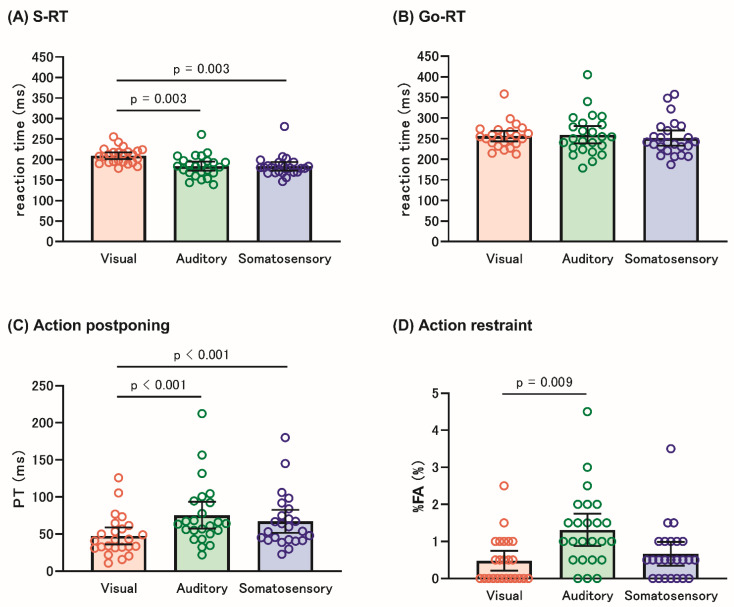
Behavioral data of the SRT and GNT in each modality. The top of the bar and whisker plot represents the mean value and 95% confidence interval (CI), respectively. The orange, green, and blue blocks indicate the visual, auditory, and somatosensory modalities, respectively. (**A**) The S-RT in the SRT was shorter in the visual modality than in the auditory and somatosensory modalities, while (**B**) the Go-RT in the GNT did not differ among the three modalities. (**C**,**D**) show the AP and AR for each modality, respectively. AP was assessed using PT, which is the difference between the S-RT and Go-RT, and AR was assessed using %FA. We demonstrated a shorter PT in the visual modality than in the other modalities and lower %FA in the visual modality than in the auditory modality. S-RT, simple reaction time; SRT, simple reaction task; Go-RT, Go reaction time; GNT, Go/No-go task; AP, action postponing; AR, action restraint; PT, postponing time; %FA, % false alarm.

Table 1 and Figure 4A–D show the behavioral data for SRT and GNT in each sensory modality.

The Friedman test revealed significant differences among the sensory modalities for S-RT (X^2^ = 23.524, *p* < 0.001), Go-RT (X^2^ = 9.238, *p* = 0.01), PT (X^2^ = 18.952, *p* < 0.001), %FA (X^2^ = 9.072, *p* = 0.011). Post hoc tests showed that the S-RT was longer in the visual modality than in the auditory (*p* = 0.003) and somatosensory modalities (*p* = 0.003), and the PT was significantly shorter in the visual modality than in the other modalities (*p* < 0.001). Moreover, the visual modality had a significantly lower %FA (*p* = 0.009) than the auditory modality. However, there were no significant differences among the three modalities in the Go-RT. Sex differences in PT and %FA are shown in the Supplementary Materials Figures S1 and S2.

### 3.2. AP-Related ERP Components

**Figure 5:**
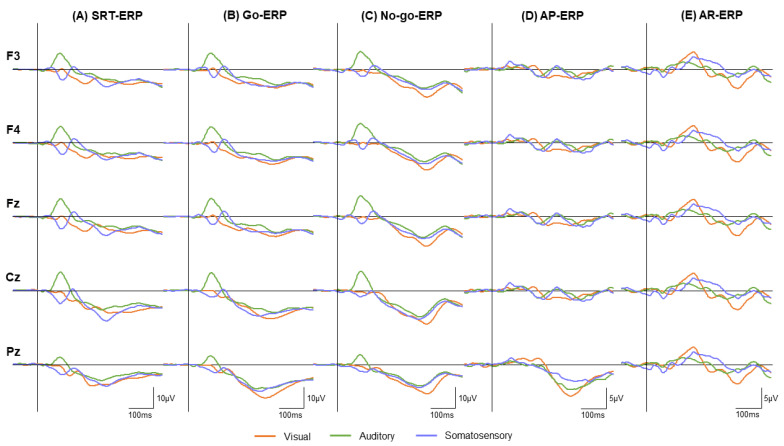
Grand-averaged SRT-, Go-, No-go-, AP-, and AR-ERP in each modality. The orange, green, and blue lines represent the visual, auditory, and somatosensory modalities, respectively. Each column shows (**A**) signal-locked ERP in the SRT, (**B**) Go signal-locked ERP in the GNT, and (**C**) No-go-signal-locked ERP in the GNT at five electrodes. (**D**) the AP-ERP, which calculates the waveform difference by subtracting the SRT-ERP from Go-ERP. (**E**) the AR-ERP, which calculates the waveform difference by subtracting the Go-ERP from No-go-ERP at each electrode. The present study obtained main significance findings that the AP-N2 latency at F3, F4, and Fz was significantly shorter in the visual modality than in the auditory (*p* = 0.003) and somatosensory modalities (*p* = 0.001) (**D**). Additionally, the AR-N2 amplitude at F4 was larger in the visual modality than in the auditory modality (*p* = 0.036) and the P3 latency at Cz was longer in the visual modality than in the auditory modality (*p* = 0.042) (**E**). AP, action postponing; AR, action restraint; SRT, simple reaction task; ERP, event-related potential; SRT-ERP, signal-locked waveforms in SRT; Go-ERP, Go signal-locked waveforms; No-go-ERP, No-go-signal-locked waveforms.

Figure 5 shows the grand-averaged waveforms for the SRT-, Go-, No-go-, AP-, and AR-ERP at five electrode positions (F3, F4, Fz, Cz, and Pz) for the visual, auditory, and somatosensory modalities. Table 2 shows the latency and amplitude of the N2 and P3 components in the AP- and AR-ERP at each electrode. The latency and amplitude of the SRT-, Go-, and No-go-ERP components for the three modalities are shown in the Supplementary Materials Table S2.

**Figure 6:**
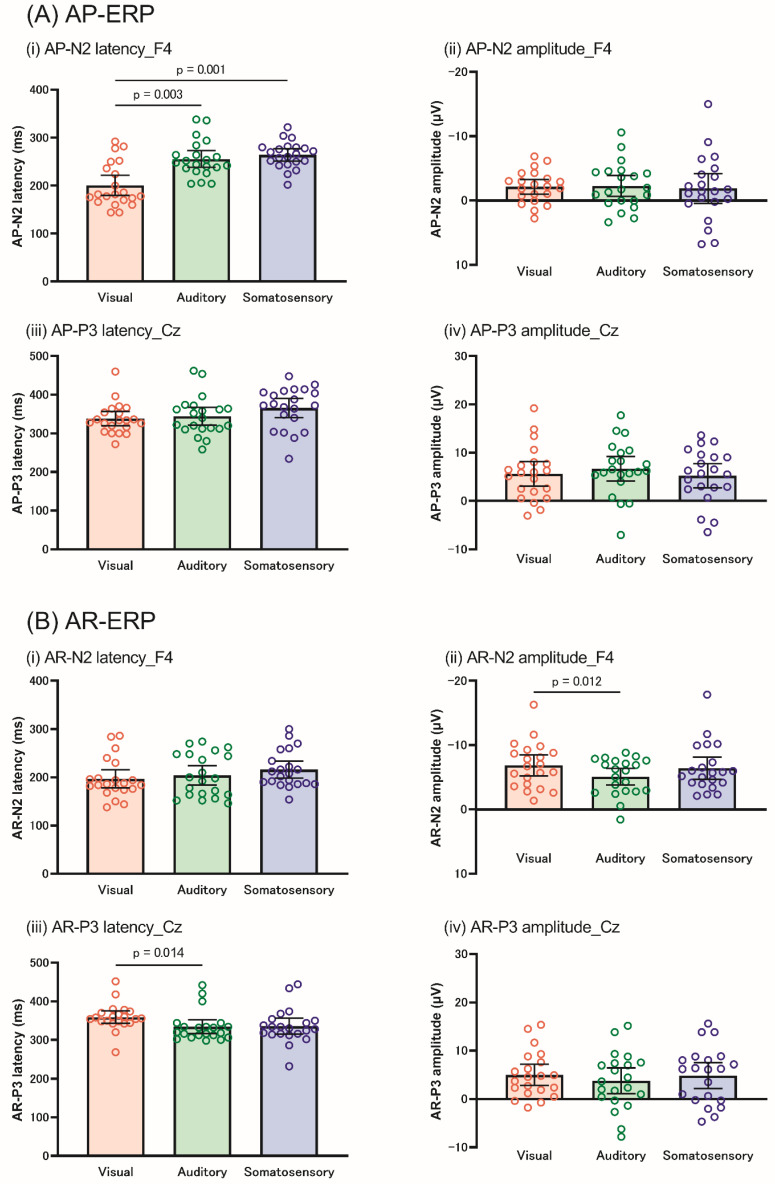
The differences in the AP-ERP (**A**) and AR-ERP (**B**) among each sensory modality. The orange, green, and blue blocks indicate the visual, auditory, and somatosensory modalities, respectively. (**A**) shows that AP-N2 latency was shorter in the visual modality than in the auditory and somatosensory modalities (i), while AP-N2 amplitude (ii), AP-P3 latency (iii), and amplitude (iv) were not significant. (**B**) AR-N2 amplitude was larger in the visual modality than in the auditory modality (ii). AR-N2 latency (i), AR-P3 latency (iii), and amplitude (iv) did not differ among the sensory modalities. The N2 and P3 components indicate the data from F4 and Cz, respectively. AP, action postponing; AR, action restraint; ERP, event-related potential.

For AP-N2, the Friedman test revealed that N2 latency differed among the sensory modalities at F3 (X^2^ = 13.238, *p* = 0.001), F4 (X^2^ = 10.289, *p* = 0.006), and Fz (X^2^ = 11.143, *p* = 0.004). In all the electrodes, the N2 latency was significantly shorter in the visual modality than in the auditory (*p* = 0.003) and somatosensory modalities (*p* = 0.001) (Figure 6A). In addition, two-way repeated-measures ANOVA revealed significant main effects of the electrode on N2 amplitude (F [2, 40] = 6.069, *p* = 0.005, η*p*^2^ = 0.233), while no significant effects in the modality and the interaction between the modality and electrode were observed.

For AP-P3, the Friedman test revealed a significant difference in the modality for P3 latency at Cz (X^2^ = 9.732, *p* = 0.008); however, the post hoc test revealed no significance. Two-way repeated-measures ANOVA showed that the P3 amplitude did not show any significant effect or interaction.

### 3.3. AR-Related ERP Components

The Friedman test revealed no significant differences among the sensory modalities for AR-N2 latency at any electrode. In contrast, the AR-N2 amplitude showed significant differences between the modalities for the AR-N2 amplitude at F3 (X^2^ = 7.238, *p* = 0.027) and F4 (X^2^ = 7.238, *p* = 0.027), but not at Fz. The post hoc test revealed a larger N2 amplitude at F4 in the visual modality than in the auditory modality (*p* = 0.036). Figure 6B shows the difference in N2 amplitude among the three modalities at each electrode.

Additionally, the Friedman test showed significant differences in the modality for P3 latency at Cz (X^2^ =10.627, *p* = 0.005) and Pz (X^2^ = 10.296, *p* = 0.006). Post hoc tests revealed that the P3 latency at Cz was longer in the visual modality than in the auditory modality (*p* = 0.042), whereas Pz did not differ significantly among the sensory modalities.

### 3.4. Relationship between the Behavioral and Neurophysiological Data for AP and AR

**Figure 7:**
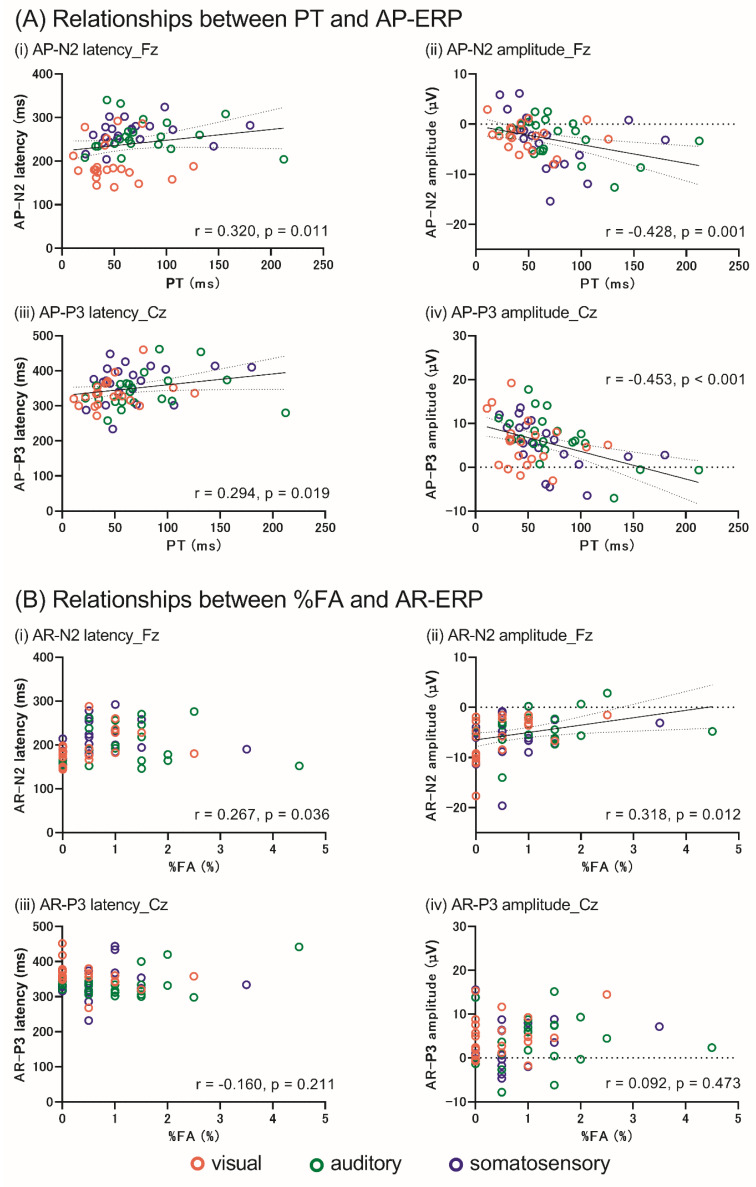
Relationships between the behavioral and neurophysiological data. The orange, green, and blue circles represent the data from the visual, auditory, and somatosensory modalities, respectively. (**A**) Relationship between the PT, AP-N2, and P3. PT was significantly associated with the N2 and P3 latencies and amplitudes (i–iv). (**B**) shows the relationship between %FA and AR-N2 and -P3. There was a significant positive correlation between %FA and AR-N2 amplitude (ii). In contrast, N2 latency (i), P3 latency (iii), and amplitude (iv) showed no significant correlations. The N2 and P3 components indicate the data from Fz and Cz, respectively. AP, action postponing; AR, action restraint; ERP, event-related potential; PT, postponing time; %FA, % false alarm.

Supramodal correlation analysis revealed that the PT was significantly associated with AP-N2 latencies (F3: r = 0.322, *p* = 0.011; F4: r = 0.322, *p* = 0.011; Fz: r = 0.320, *p* = 0.011) and amplitudes (F3: r = −0.394, *p* = 0.002; F4: r = −0.434, *p* < 0.001; Fz: r = −0.428, *p* = 0.001). Additionally, there were significant correlations between the PT and AP-P3 latencies (Cz: r = 0.294, *p* = 0.019; Pz: r = 0.328, *p* = 0.009) and amplitudes (Cz: r = −0.453, *p* < 0.001; Pz: r = −0.335, *p* = 0.007). Moreover, significant relationships were observed between the %FA and the AR-N2 amplitudes at F3 (r = 0.337, *p* = 0.007) and Fz (r = 0.318, *p* = 0.012), but not at F4. The AR-N2 latencies, AR-P3 latencies, and amplitudes were not significantly correlated with the %FA. Figure 7 shows the relationship between the behavioral (PT [A], %FA [B]) and neurophysiological (AP-, AR-N2, and -P3) data. The results of the correlation analysis between the behavioral and neurophysiological data related to the AP and AR in each sensory modality are presented in the Supplementary Materials Tables S3 and S4.

## 4. Discussion

The present study examined whether sensory modalities alter AP and AR. The main findings of the present study were (1) shorter PT in the visual modality than in the auditory and somatosensory modalities, and (2) lower %FA in the visual modality than in the auditory modality.

Proactive inhibition consists of AP and AR. Previously, these components were evaluated using the GNT paradigm; however, research has scarcely assessed them separately. Nearly all the previous studies have used the Go-RT to assess both AP and AR, reporting inconsistent results [14,16,17,36]. One of the explanations for this would be that Go-RT reflects not only the time to slow the onset of response but also S-RT to each sensory stimulus. The S-RT differed across the sensory modalities, as shown in the present results. Therefore, the present study used the assessment by subtracting S-RT from Go-RT to solve this issue.

Additionally, considering the comparison between the three modalities, the paradigm taking into consideration the SRC is preferred to report the SRC effect in visual and auditory GNT [16,20]. Therefore, we adopted the present paradigm to avoid the SRC effect, in which the Go and No-go signals were presented to the right and left sides of the participants, respectively, and they responded with their right hand to the Go signal in all theGNT. Previous studies mainly compared two sensory modalities. To the best of our knowledge, this is the first study to directly compare the three modalities by solving these methodological issues of previous studies and to find a difference in the AP and AR across sensory modalities.

### 4.1. Sensory Modality-Related AP

The present results showed the shortest PT in the visual modality compared to the auditory and somatosensory modalities, while the Go-RT was not different among the three modalities, consistent with our hypothesis. Taken together with our previous result that PT induced by AC (using SST) was shorter in the visual modality than those in the auditory and somatosensory modalities [13], it is suggested that the visual signal is the weakest trigger to occur AP among visual, auditory and somatosensory modality when driving not only AC but also AR. Regarding Go-RT, the present results were consistent with those of previous studies that investigated the effect of sensory modality on proactive inhibition [6,14,17]. To the best of our knowledge, this is the first study to show the difference in AP induced by AR among sensory modalities.

The shorter PT in the visual modality could be explained by the faster neural processing for premotor inhibitory processes or conflict monitoring induced by anticipating the presence of a No-go signal, followed by shorter AP-N2 latency in the visual modality. Previous studies have reported that N2 reflects premotor inhibitory processes [15,21] and conflict monitoring [22,23]; moreover, this latency shows the speed of the conflict monitoring [37,38]. N2 latency is reportedly delayed when task complexity is increased [21,39] and when the relative frequency of the No-go signal is increased [40]. Therefore, a shorter AP-N2 latency would show faster neural processing for conflict monitoring in the visual modality than in other modalities. Proactive inhibition, which includes AP and AR, is goal-directed and needs to be mediated by WM to manage the information concerning the time and means of implementing inhibition [12]. In addition, several previous studies have reported higher visual WM compared to other sensory modalities [29,30]. Considering these results, higher visual WM might enable a faster neural processing speed for conflict monitoring, which might involve shorter PT in the visual modality.

Additionally, supramodal correlation analysis revealed that the AP-N2 amplitudes were negatively correlated with the PT; however, there was no difference among the three sensory modalities, suggesting that the AP-N2 amplitude is a supramodal common neural activity, and one of the explained indices for the amount of conflict monitoring for Go signal in the GNT irrespective of the sensory modalities. Previous studies have reported that No-go-N2 amplitudes increase when a greater amount of neural resources are required for conflict monitoring [32,41]. Conflict monitoring refers to the cognitive resources required to deal with distraction-induced interference [42]. Based on these previous studies, smaller AP-N2 amplitudes would require lower cognitive resources to implement correct Go responses by suppressing distracted interference induced by the No-go signal. Thus, the present results, in which the smaller the AP-N2 amplitudes the shorter PT, indicated that the participants with lower neural activity for conflict monitoring could quickly respond to the Go signal in the GNT irrespective of the sensory modality.

Similar to AP-N2, the AP-P3 amplitude was negatively correlated with PT, which shows that larger AP-P3 amplitudes are associated with shorter PT in the GNT. This could be explained by the attentional resource for response execution for the Go signal involved in the AP in the GNT. P3 has been reported to reflect not only the evaluation of inhibition but also the amount of attentional resources [43,44]. Our previous study also showed that larger AP-P3 amplitudes, which indicate the attention to response execution, were associated with the response time in the SST to assess reactive inhibition [13]. Therefore, larger AP-P3 amplitudes in the GNT indicate greater attention to the response execution to the Go signal in the GNT, which resulted in a shorter PT when the task differed from the previous study.

### 4.2. Sensory Modality-Related AR

The present results revealed a lower %FA in the visual modality than in the auditory modality. This result indicates that the visual modality is more powerful in triggering AR than the auditory modality. Unlike the present findings, previous studies have reported no significant difference in %FA between visual and auditory modalities and the lower %FA in the somatosensory modality compared with the visual modalities [6,14,16]. One potential explanation for this difference could be the SRC effect, which refers to the phenomenon in which some mappings between a particular set of stimuli and responses allow for better performance than other mappings [18,19]. We used a GNT paradigm considering the SRC effect that was not the case in the previous studies and that could involve lower %FA in visual modality.

The reason for the lower %FA in the visual modality can be explained by the higher neural resources for conflict monitoring induced by the presence of the No-go signals. Previous GNT studies have reported that No-go-N2 amplitude (which is termed “AR-N2” in the present study) was larger in the low %FA group than in the high %FA group [15]. Other studies have demonstrated that a larger No-go-N2 amplitude indicates the need for high neural resources for pre-response conflict, leading to a precise response [31,32]. These findings suggest that a more efficient AR is caused by a larger AR-N2 amplitude. Since the AR-N2 component is known to represent conflict monitoring previously, the amplitude would reflect the amount of neural resources needed for conflict monitoring, and an increasing amount of resources would enable precise responses for the No-go signal. The present results demonstrated a significantly lower error rate and a larger AR-N2 amplitude in the visual modality than in the auditory modality. Based on these results, the visual modality is more powerful than the auditory modality in driving neural activity for conflict monitoring, which enables a higher accuracy in the visual modality. Interestingly, the supramodal correlation analysis demonstrated that the %FA is positively associated with the AR-N2 amplitude (Figure 7B(ii)). These results indicate that the AR-N2 amplitude suggests the involvement of supramodal common neural processing in conflict monitoring during the GNT, and explains not only the difference in the AR among the three sensory modalities but also the variability of supramodal AR. Additionally, higher WM in the visual modality would also be associated with higher accuracy in the visual modality, similar to AP. A previous study demonstrated that higher WM results in flexible adjustment of conflict monitoring [42]. This flexible adjustment is suggested to enable better preparation for rapid and precise responses [45]. Considering the higher visual WM in previous studies [29,30], visual modality could adjust and intensify conflict monitoring resulting in a lower error rate.

### 4.3. Limitations

There are three limitations to the present study. First, the laterality of sensory receptors could be involved in the results. To eliminate the effects of the SRC, the direction of the stimulus and response were congruent. Thus, sensory stimuli were applied to the unilateral receptors in the auditory and somatosensory modalities, and to the bilateral receptors in the visual modality for response execution/inhibition. Therefore, we cannot exclude the possibility that the experimental setting could have affected our results. Second, this is the first study to assess the AP and its related neural processing by subtracting the S-RT and SRT-ERP from the Go-RT and Go-ERP, respectively. Therefore, further research is needed to confirm the validity of this methodology for AP assessment. Third, we cannot refer to the scalp topographic differences in AP and AR among sensory modalities. The present study measured EEG signals from 9 electrodes to assess AP- and AR-related neural processing among sensory modalities. However, it would have been mandatory to employ 64 electrodes to perform source localization analyses. Therefore, in order to identify the scalp topographic differences among sensory modalities, further study is needed.

## 5. Conclusions

The present results demonstrated that (1) shorter PT resulted in faster conflict monitoring-related neural processing in the visual modality than in the auditory and somatosensory modalities and (2) better AR resulted in intense conflict monitoring-related neural activation in the visual modalities compared to the auditory and somatosensory modalities. These findings indicate that the visual modality can drive higher AR with lower AP compared to other sensory modalities. Additionally, it is preferable to evaluate AP and AR separately when examining proactive inhibition because the sensory modality is involved in driving both AP and AR.

## Figures and Tables

**Table 1:** Behavioral data for each modality.

		Visual	Auditory	Somatosensory
Simple reaction task				
	S-RT (ms)	208.93 ± 3.54 *	183.81 ± 5.64	185.27 ± 5.56
Go/No-go task				
	Go-RT (ms)	257.92 ± 6.71	262.10 ± 11.07	252.71 ± 9.57
	PT (ms)	48.99 ± 6.13 *	78.29 ± 9.79	67.44 ± 8.38
	%FA	0.48 ± 0.14 ^†^	1.21 ± 0.22	0.71 ± 0.17

Mean ± SEM; S-RT, simple reaction time; Go-RT, reaction time for Go signal; PT, postponing time; FA, false alarms. Note. The asterisk (*) indicates a significant difference compared with the auditory and somatosensory modalities. The dagger (^†^) indicates a significant difference compared with the auditory modality.

**Table 2:** Neurophysiological data for each modality.

	Visual		Auditory		Somatosensory	
	Latency (ms)	Amplitude (µV)	Latency (ms)	Amplitude (µV)	Latency (ms)	Amplitude (µV)
AP-ERP						
N2						
F3	196.67 ± 9.76 *	−2.03 ± 0.57	256.48 ± 8.38	−2.50 ± 0.74	265.70 ± 6.41	−2.10 ± 1.16
F4	195.62 ± 10.14 *	−2.23 ± 0.52	255.71 ± 8.29	−2.27 ± 0.78	265.20 ± 6.27	−1.90 ± 1.17
Fz	195.90 ± 10.04 *	−2.41 ± 0.60	257.14 ± 8.25	−2.79 ± 0.85	265.60 ± 6.39	−3.17 ± 1.23
P3						
Cz	337.71 ± 8.79	5.61 ± 1.22	344.38 ± 11.14	6.68 ± 1.23	365.62 ± 11.77	5.20 ± 1.21
Pz	335.52 ± 7.57	8.08 ± 1.15	347.05 ± 11.50	8.55 ± 0.90	354.29 ± 12.78	6.10 ± 0.95
AR-ERP						
N2						
F3	195.24 ± 8.26	−5.36 ± 0.85	203.08 ± 9.99	−3.74 ± 0.66	213.14 ± 7.92	−5.52 ± 1.05
F4	196.86 ± 8.98	−6.86 ± 0.77 ^†^	206.80 ± 9.82	−5.22 ± 0.64	216.10 ± 8.41	−6.41 ± 0.82
Fz	193.62 ± 7.92	−6.28 ± 0.96	204.00 ± 9.54	−4.17 ± 0.78	211.90 ± 8.17	−5.52 ± 0.95
P3						
Cz	359.14 ± 7.61 ^†^	5.01 ± 1.04	334.38 ± 8.55	3.76 ± 1.28	335.81 ± 9.91	4.82 ± 1.27
Pz	365.14 ± 7.24	2.88 ± 1.03	344.38 ± 8.67	1.03 ± 0.89	340.76 ± 9.66	1.68 ± 0.86

Mean ± SEM AP, action postponing; AR, action restraint; ERP, event-related potential; AP-ERP, waveform of simple reaction task (SRT)-ERP subtracted from Go-ERP in Go/No-go task (GNT), AR-ERP; waveform of Go-ERP subtracted from No-go-ERP. The asterisk (*) indicates a significant difference compared with the auditory and somatosensory modalities. The dagger (^†^) indicates a significant difference compared with the auditory modality.

## Data Availability

The data presented in this study are available on request from the corresponding author.

## Supplementary Materials

The following supporting information can be downloaded at: https://www.mdpi.com/article/10.3390/brainsci12111530/s1, Figure S1: Sex difference of the postponing time (PT) in each modality; Figure S2: Sex difference of the percentage of false alarm (%FA) in each modality; Table S1: Sex differences in the behavioral data for each modality; Table S2: Neurophysiological data for each modality; Table S3: Relationship between the behavioral and neurophysiological data related to action postponing in each modality; Table S4: Relationship between the behavioral and neurophysiological data for action restraint in each modality.

## References

[1] Matzke D., Verbruggen F., Logan G.D. (2018). The Stop-Signal Paradigm. Stevens’ Handbook of Experimental Psychology and Cognitive Neuroscience.

[2] Bari A., Robbins T.W. (2013). Inhibition and impulsivity: Behavioral and neural basis of response control. Prog. Neurobiol..

[3] Hannah R., Aron A.R. (2021). Towards real-world generalizability of a circuit for action-stopping. Nat. Rev. Neurosci..

[4] Jahfari S., Verbruggen F., Frank M.J., Waldorp L.J., Colzato L., Richard Ridderinkhof K., Forstmann B.U. (2012). How Preparation Changes the Need for Top–Down Control of the Basal Ganglia When Inhibiting Premature Actions. J. Neurosci..

[5] Zandbelt B.B., Bloemendaal M., Neggers S.F.W., Kahn R.S., Vink M. (2013). Expectations and violations: Delineating the neural network of proactive inhibitory control. Hum. Brain Mapp..

[6] Walther S., Goya-Maldonado R., Stippich C., Weisbrod M., Kaiser S. (2010). A supramodal network for response inhibition. Neuroreport.

[7] Zhang D., Ding H., Wang X., Qi C., Luo Y. (2015). Enhanced response inhibition in experienced fencers. Sci. Rep..

[8] Leunissen I., Van Steenkiste M., Heise K.-F., Monteiro T.S., Dunovan K., Mantini D., Coxon J.P., Swinnen S.P. (2022). Effects of beta-band and gamma-band rhythmic stimulation on motor inhibition. iScience.

[9] Raud L., Thunberg C., Huster R.J. (2022). Partial response electromyography as a marker of action stopping. Elife.

[10] Braver T.S. (2012). The variable nature of cognitive control: A dual mechanisms framework. Trends Cogn. Sci..

[11] Jaffard M., Longcamp M., Velay J.-L., Anton J.-L., Roth M., Nazarian B., Boulinguez P. (2008). Proactive inhibitory control of movement assessed by event-related fMRI. Neuroimage.

[12] Meyer H.C., Bucci D.J. (2016). Neural and behavioral mechanisms of proactive and reactive inhibition. Learn. Mem..

[13] Ikarashi K., Sato D., Fujimoto T., Edama M., Baba Y., Yamashiro K. (2022). Response Inhibitory Control Varies with Different Sensory Modalities. Cereb. Cortex.

[14] Bodmer B., Beste C. (2017). On the dependence of response inhibition processes on sensory modality. Hum. Brain Mapp..

[15] Falkenstein M., Hoormann J., Hohnsbein J. (1999). ERP components in Go/Nogo tasks and their relation to inhibition. Acta Psychol..

[16] Lien M.-C., Pedersen L., Proctor R.W. (2016). Stimulus-response correspondence in go-nogo and choice tasks: Are reactions altered by the presence of an irrelevant salient object?. Psychol. Res..

[17] Yamashiro K., Yamazaki Y., Siiya K., Ikarashi K., Baba Y., Otsuru N., Onishi H., Sato D. (2021). Modality-specific improvements in sensory processing among baseball players. Sci. Rep..

[18] Alluisi E.A., Warm J.S., Proctor R.W., Reeve T.G. (1990). Things That Go Together: A Review of Stimulus-Response Compatibility and Related Effects. Advances in Psychology.

[19] Proctor R.W., Vu K.-P.L. (2006). Stimulus-Response Compatibility Principles: Data, Theory, and Application.

[20] Dolk T., Liepelt R. (2018). The Multimodal Go-Nogo Simon Effect: Signifying the Relevance of Stimulus Features in the Go-Nogo Simon Paradigm Impacts Event Representations and Task Performance. Front. Psychol..

[21] Gajewski P.D., Falkenstein M. (2013). Effects of task complexity on ERP components in Go/Nogo tasks. Int. J. Psychophysiol..

[22] Randall W.M., Smith J.L. (2011). Conflict and inhibition in the cued-Go/NoGo task. Clin. Neurophysiol..

[23] Smith J.L. (2011). To go or not to go, that is the question: Do the N2 and P3 reflect stimulus- or response-related conflict?. Int. J. Psychophysiol..

[24] Bokura H., Yamaguchi S., Kobayashi S. (2001). Electrophysiological correlates for response inhibition in a Go/NoGo task. Clin. Neurophysiol..

[25] Bruin K.J., Wijers A.A., van Staveren A.S. (2001). Response priming in a go/nogo task: Do we have to explain the go/nogo N2 effect in terms of response activation instead of inhibition?. Clin. Neurophysiol..

[26] Kropotov J.D., Ponomarev V.A., Pronina M., Jäncke L. (2017). Functional indexes of reactive cognitive control: ERPs in cued go/no-go tasks. Psychophysiology.

[27] Nieuwenhuis S., Yeung N., Cohen J.D. (2004). Stimulus modality, perceptual overlap, and the go/no-go N2. Psychophysiology.

[28] Fogarty J.S., Barry R.J., Steiner G.Z. (2020). The First 250 ms of Auditory Processing: No Evidence of Early Processing Negativity in the Go/NoGo Task. Sci. Rep..

[29] Schifferstein H.N.J., Smeets M.A.M., Postma A. (2010). Comparing location memory for 4 sensory modalities. Chem. Senses.

[30] Wolff M.J., Kandemir G., Stokes M.G., Akyürek E.G. (2020). Unimodal and Bimodal Access to Sensory Working Memories by Auditory and Visual Impulses. J. Neurosci..

[31] Iannaccone R., Hauser T.U., Staempfli P., Walitza S., Brandeis D., Brem S. (2015). Conflict monitoring and error processing: New insights from simultaneous EEG-fMRI. Neuroimage.

[32] Nieuwenhuis S., Yeung N., van den Wildenberg W., Ridderinkhof K.R. (2003). Electrophysiological correlates of anterior cingulate function in a go/no-go task: Effects of response conflict and trial type frequency. Cogn. Affect. Behav. Neurosci..

[33] Lakens D., Caldwell A.R. (2021). Simulation-Based Power Analysis for Factorial Analysis of Variance Designs. Adv. Methods Pract. Psychol. Sci..

[34] Bannbers E., Gingnell M., Engman J., Morell A., Comasco E., Kask K., Garavan H., Wikström J., Sundström Poromaa I. (2012). The effect of premenstrual dysphoric disorder and menstrual cycle phase on brain activity during response inhibition. J. Affect. Disord..

[35] Mannarelli D., Pauletti C., Petritis A., Delle Chiaie R., Currà A., Trompetto C., Fattapposta F. (2020). Effects of Cerebellar tDCS on Inhibitory Control: Evidence from a Go/NoGo Task. Cerebellum.

[36] Falkenstein M., Yordanova J., Kolev V. (2006). Effects of aging on slowing of motor-response generation. Int. J. Psychophysiol..

[37] Guo Y., Schmitz T.W., Mur M., Ferreira C.S., Anderson M.C. (2018). A supramodal role of the basal ganglia in memory and motor inhibition: Meta-analytic evidence. Neuropsychologia.

[38] Guo Z., Chen R., Liu X., Zhao G., Zheng Y., Gong M., Zhang J. (2018). The impairing effects of mental fatigue on response inhibition: An ERP study. PLoS ONE.

[39] Brydges C.R., Clunies-Ross K., Clohessy M., Lo Z.L., Nguyen A., Rousset C., Whitelaw P., Yeap Y.J., Fox A.M. (2012). Dissociable components of cognitive control: An event-related potential (ERP) study of response inhibition and interference suppression. PLoS ONE.

[40] Donkers F.C.L., van Boxtel G.J.M. (2004). The N2 in go/no-go tasks reflects conflict monitoring not response inhibition. Brain Cogn..

[41] Band G.P.H., Ridderinkhof K.R., van der Molen M.W. (2003). Speed-accuracy modulation in case of conflict: The roles of activation and inhibition. Psychol. Res..

[42] Weldon R.B., Mushlin H., Kim B., Sohn M.-H. (2013). The effect of working memory capacity on conflict monitoring. Acta Psychol..

[43] Kok A. (2001). On the utility of P3 amplitude as a measure of processing capacity. Psychophysiology.

[44] Polich J. (2007). Updating P300: An integrative theory of P3a and P3b. Clin. Neurophysiol..

[45] Leue A., Weber B., Beauducel A. (2014). How do working-memory-related demand, reasoning ability and aversive reinforcement modulate conflict monitoring?. Front. Hum. Neurosci..

