# Supplementary material for: Action Postponing and Restraint Varies among Sensory Modalities
Source: Brain Sci. 2022 Nov 11;12(11):1530. doi: 10.3390/brainsci12111530 (PMC9688532; doi:10.3390/brainsci12111530)
Supplement: Supplementary file 1 [file brainsci-12-01530-s001.zip › brainsci-2011428-supplementary.pdf]

Supplementary Material

Sex difference in the behavioral data in each modality

The present study controlled for the menstrual cycle variation in female participants to reduce the effect of sex steroid hormones on the action postponing (AP) and action restraint (AR). We examined the difference in the AP and AR between the males and females in each modality using the Mann–Whitney U test with "sex" as a between-factor. The Mann–Whitney U test revealed significant sex differences in the simple response time (S-RT) in the visual (p = 0.020) and somatosensory modalities (p = 0.024), which indicated that the S-RT in these modalities was shorter in women than in men. However, we found no significant differences in the S-RT in the auditory modality and the postponing time (PT), the percentages of the false alarms (%FA) and omission errors (%OE) for each sensory modality between the men and women. Therefore, no sex differences were observed in the AP and AR for each sensory modality.

Table S1. Sex differences in the behavioral data for each modality

	Visual	Auditory	Somatosensory
Simple reaction task			
S-RT (ms)			
Male	216.17 ± 5.23	189.28 ± 9.55	193.84 ± 9.50
Female	200.97 ± 3.41*	177.80 ± 5.41	175.85 ± 3.91*
Go/No-go task			
Go-RT (ms)			
Male	264.17 ± 11.06	270.83 ± 17.57	263.14 ± 12.87
Female	251.05 ± 6.93	252.50 ± 13.24	241.24 ± 14.05
PT (ms)			
Male	48.01 ± 9.69	81.55 ± 15.98	69.30 ± 10.51
Female	50.07 ± 7.79	74.70 ± 11.51	65.39 ± 13.86
% FA			
Male	0.55 ± 0.25	1.45 ± 0.39	0.91 ± 0.29
Female	0.40 ± 0.15	0.95 ± 0.17	0.50 ± 0.15
% OE			
Male	0.23 ± 0.12	0.18 ± 0.12	0.55 ± 0.25
Female	0.05 ± 0.05	0.10 ± 0.07	0.10 ± 0.10

Mean±SEM; S-RT, simple reaction time; Go-RT, reaction time for Go signal; PT, postponing time; FA, false alarms; OE, omission errors. The asterisk (*) indicates a significant difference compared to men.

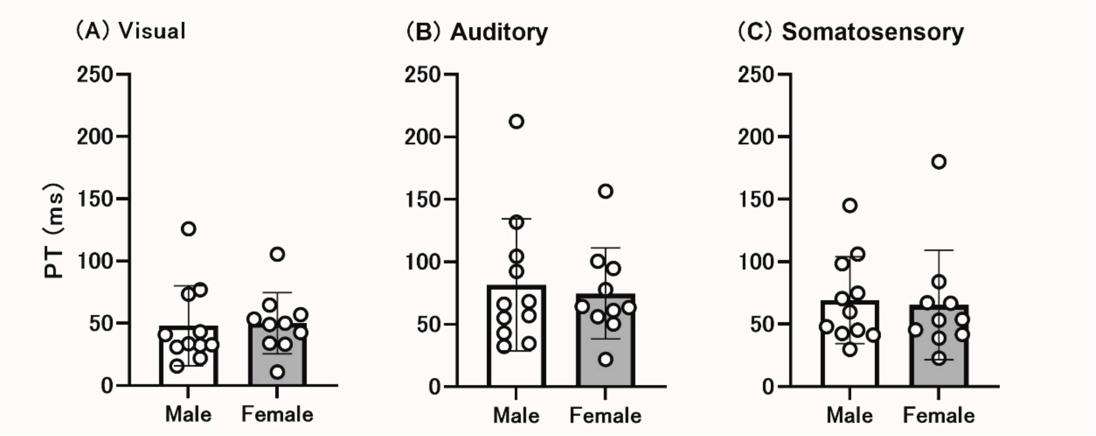

Figure S1. Sex difference of the postponing time (PT) in each modality

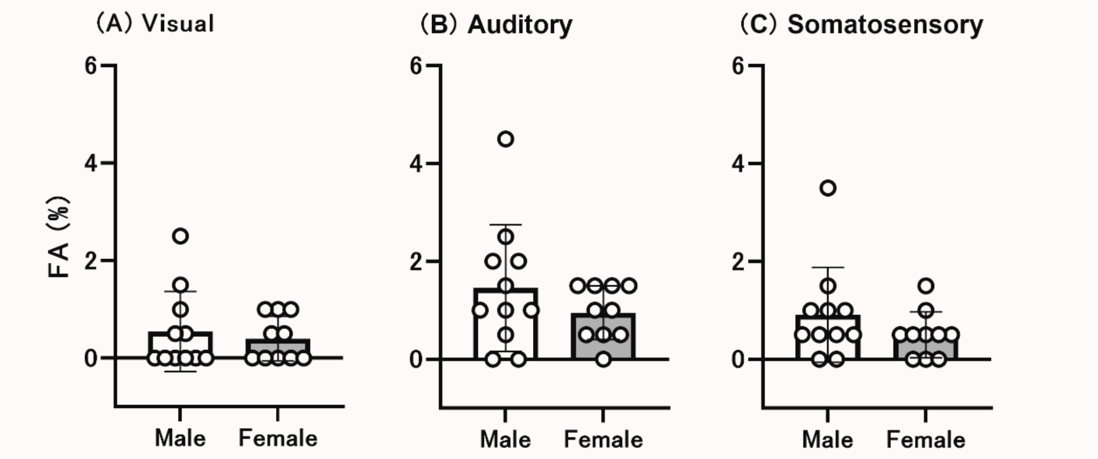

Figure S2. Sex difference of the percentage of false alarm (%FA) in each modality

Simple reaction task (SRT-), Go- and No-go-event related potential (ERP) components in each modality

Table S2 shows the latency and amplitude of the SRT-, Go-, and No-go-ERP components for the three modalities. N1 was extracted at F3, Fz, and F4 as the maximum negative values in the time window of 120–250 ms and 60–120 ms in the visual and auditory modalities, respectively. P100 (60–120 ms) and N140 (110–180 ms) were extracted as the maximum positive and negative values, respectively, at F3, Fz, and F4 in the somatosensory modality. P300 was extracted at Cz and Pz as the maximum positive value in the time window of 200–500 ms for all the modalities.

Relationship between the behavioral and neurophysiological data in AP and AR

Table S3 shows the results of the correlation analysis between the behavioral and neurophysiological data related to the AP in each sensory modality. For the auditory modality, correlation analysis revealed that there were significant negative relationships between the PT and the AP-P3 amplitude at Cz (r = -0.582, p = 0.006). For the somatosensory modality, PT was significantly correlated with the AP-N2 amplitude at F3 (r = -0.594, p = 0.005), F4 (r = -0.788, p < 0.001), Fz (r = -0.677, p = 0.001), P3 amplitude Cz (r = -0.743, p < 0.001), and Pz (r = -0.717, p < 0.001). The visual modality showed no significant relationships.

Table S4 shows the results of the correlation analysis between the behavioral and neurophysiological data in the AR for each sensory modality. For the visual modality, there were significant relationships between the %FA and AR-N2 amplitude at F3 (r = 0.611, p = 0.003), Fz (r = 0.598, p = 0.004), and P3 latency at Pz (r = -0.639, p = 0.002). The auditory and somatosensory modalities showed no significant relationships.

Table S2. Neurophysiological data for each modality

	SRT-ERP		Go-ERP		No-go-ERP	
	Latency (ms)	Amplitude (μV)	Latency (ms)	Amplitude (μV)	Latency (ms)	Amplitude (μV)
Visual						
N1						
F3	117.43 ± 6.18	0.36 ± 0.60	120.29 ± 6.37	0.07 ± 0.51	157.33 ± 5.01	-0.16 ± 0.53
F4	118.76 ± 5.93	0.31 ± 0.61	119.62 ± 7.11	0.10 ± 0.50	156.95 ± 5.42	-1.06 ± 0.63
Fz	118.76 ± 6.14	0.31 ± 0.64	121.62 ± 6.80	0.12 ± 0.50	157.62 ± 5.60	-1.08 ± 0.58
P300						
Cz	297.24 ± 12.94	12.13 ± 1.06	324.48 ± 10.93	16.00 ± 1.35	342.29 ± 7.67	17.43 ± 1.16
Pz	292.67 ± 13.55	12.17 ± 0.76	312.86 ± 9.79	17.49 ± 1.02	342.67 ± 6.77	15.18 ± 0.93
Auditory						
N1						
F3	90.57 ± 2.28	-8.55 ± 0.82	89.90 ± 2.92	-8.89 ± 0.64	92.19 ± 2.47	-9.36 ± 0.80
F4	91.43 ± 2.27	-8.96 ± 0.81	90.86 ± 2.51	-8.83 ± 0.63	91.90 ± 2.70	-10.11 ± 0.82
Fz	91.81 ± 2.22	-9.48 ± 0.90	92.38 ± 2.35	-9.58 ± 0.64	90.95 ± 2.20	-10.65 ± 0.86
P300						
Cz	266.38 ± 10.90	10.26 ± 0.93	314.76 ± 14.99	12.75 ± 1.53	327.14 ± 9.69	14.16 ± 1.22
Pz	257.81 ± 10.49	9.19 ± 0.75	314.19 ± 13.28	13.81 ± 1.18	325.52 ± 10.20	11.77 ± 0.89
Somatosensory						
P100						
F3	100.00 ± 2.44	5.55 ± 0.59	98.67 ± 2.93	4.34 ± 0.65	98.48 ± 2.57	3.93 ± 0.52
F4	97.43 ± 2.33	6.12 ± 0.52	98.57 ± 3.06	5.33 ± 0.54	96.67 ± 2.56	3.53 ± 0.59
Fz	95.62 ± 2.48	6.63 ± 0.61	96.57 ± 3.30	4.86 ± 0.64	95.62 ± 2.38	3.80 ± 0.59
N140						
F3	144.86 ± 3.03	-2.08 ± 1.07	140.76 ± 3.37	-2.79 ± 0.78	148.38 ± 4.18	-3.08 ± 0.72
F4	146.38 ± 3.41	-3.03 ± 0.97	140.59 ± 3.25	-3.03 ± 0.75	140.86 ± 4.69	-3.53 ± 0.77
Fz	147.05 ± 3.58	-3.38 ± 1.10	141.90 ± 3.24	-3.88 ± 0.87	144.10 ± 3.95	-3.63 ± 0.83

P300						
Cz	278.38 ± 9.32	14.13 ± 1.28	290.00 ± 10.76	15.21 ± 1.45	314.95 ± 9.67	15.73 ± 1.35
Pz	274.10 ± 8.57	12.02 ± 0.90	292.48 ± 10.11	14.70 ± 1.19	315.90 ± 9.36	12.51 ± 0.94

Mean±SD; SRT-ERP, event-related potential in simple reaction task; Go-ERP, event-related potential for go signal; No-go-ERP, event-related potential for No-go signal.

Table S3. Relationship between the behavioral and neurophysiological data related to action postponing in each modality

	PT (ms)					
	Visual		Auditory		Somatosensory	
	r	p	r	p	r	p
AP-ERP						
N2 latency (ms)						
F3	-0.139	0.549	0.119	0.608	0.456	0.038
F4	-0.111	0.631	0.136	0.557	0.437	0.048
Fz	-0.118	0.609	0.133	0.565	0.412	0.064
N2 amplitude (μV)						
F3	-0.386	0.084	-0.400	0.072	-0.594**	0.005
F4	-0.390	0.081	-0.292	0.199	-0.788**	0.000
Fz	-0.277	0.225	-0.382	0.088	-0.677**	0.001
P3 latency (ms)						
Cz	0.403	0.070	0.291	0.200	0.322	0.155
Pz	0.382	0.088	0.270	0.236	0.383	0.086
P3 amplitude (μV)						
Cz	-0.243	0.289	-0.582**	0.006	-0.743**	0.000
Pz	-0.014	0.951	-0.436	0.048	-0.717**	0.000

AP-ERP: waveform of simple reaction task (SRT)-ERP subtracted from the Go-ERP in the Go/No-go task (GNT), PT: postponing time. ** $p < 0.01$, *$p < 0.05$

Table S4. Relationship between the behavioral and neurophysiological data for action restraint in each modality

	%FA					
	Visual		Auditory		Somatosensory	
	r	p	r	p	r	p
AP-ERP						
N2 latency (ms)						
F3	0.488	0.025	0.082	0.723	0.410	0.065
F4	0.467	0.033	0.019	0.935	0.411	0.065
Fz	0.423	0.056	0.034	0.883	0.396	0.075
N2 amplitude (μV)						
F3	0.611**	0.003	0.036	0.876	0.284	0.213
F4	0.341	0.131	-0.036	0.876	-0.040	0.862
Fz	0.598**	0.004	0.097	0.677	0.109	0.639
P3 latency (ms)						
Cz	-0.437	0.048	-0.014	0.953	0.201	0.382
Pz	-0.639**	0.002	0.106	0.647	0.318	0.160
P3 amplitude (μV)						
Cz	0.184	0.423	0.198	0.389	-0.072	0.757
Pz	0.069	0.768	0.138	0.550	0.018	0.937

AR-ERP: waveform of Go-ERP subtracted from No-go-ERP; FA: false alarm. ** $p < 0.01$, *$p < 0.05$
